# Partial hepatectomy for pancreatic-origin hepatic abscess with intracystic hemorrhage following severe acute pancreatitis: a case report

**DOI:** 10.3389/fmed.2026.1743146

**Published:** 2026-03-05

**Authors:** Hongji Xu, Ziyu Zhang, Yibo Yang, Jiahao Zuo, Xiang Deng

**Affiliations:** 1 Department of Abdominal Surgery, Guiqian International Hospital, Guiyang, Guizhou, China; 2 Department of Hepatobiliary and Pancreatic Surgery, General Surgery Center, First Hospital of Jilin University, Changchun, Jilin, China; 3 Changchun University of Chinese Medicine, Changchun, Jilin, China

**Keywords:** intracystic hemorrhage, pancreatic pseudocyst, pancreatic-origin hepatic abscess, partial hepatectomy, severe acute pancreatitis

## Abstract

Local complications of severe acute pancreatitis commonly include pancreatic necrosis, pancreatic pseudocysts, and peripancreatic abscesses. However, hepatic abscess secondary to pancreatitis is extremely rare. The routes of infection may occur through dissemination via the portal venous system or lymphatic system, or through direct infiltration from peripancreatic inflammatory spread. The latter, due to direct hepatic invasion by bacteria and amylase, is prone to complicated intracystic hemorrhage. This article aims to explore the management of a rare case of pancreatic-origin hepatic abscess with intracystic hemorrhage treated primarily through partial hepatectomy, summarizing therapeutic experience for such patients.

## Introduction

Hepatic abscess is a focal suppurative infection of the liver that typically arises from biliary infection, hematogenous spread, or contiguous extension from adjacent structures (1). Although modern imaging, antibiotics, and percutaneous drainage have improved outcomes, abscesses caused by uncommon etiologies remain diagnostically challenging and may lead to severe complications (2).

Pancreatic-origin hepatic abscess is an exceptionally rare entity, most often occurring in the setting of acute or chronic pancreatitis complicated by pancreatic necrosis or an infected pancreatic pseudocyst (3). Because pancreatitis and hepatic abscess share nonspecific features, such as fever, abdominal pain, and inflammatory marker elevation, delayed recognition or misdiagnosis may occur.

One proposed mechanism is direct penetration of peripancreatic necrosis or an infected pseudocyst into the hepatic capsule, allowing pathogens and pancreatic enzymes to enter the liver and potentially precipitate hemorrhagic complications if communication with the biliary tree exists. Evidence remains limited, and optimal management strategies are not well defined (4).

Here, we report a rare case of pancreatic-origin hepatic abscess with intracystic hemorrhage following severe acute pancreatitis. We highlight key diagnostic clues on contrast-enhanced CT, discuss the presumed pathophysiology, and describe a multidisciplinary management approach in which partial hepatectomy played a central role.

## Case presentation

**Figure 1:**
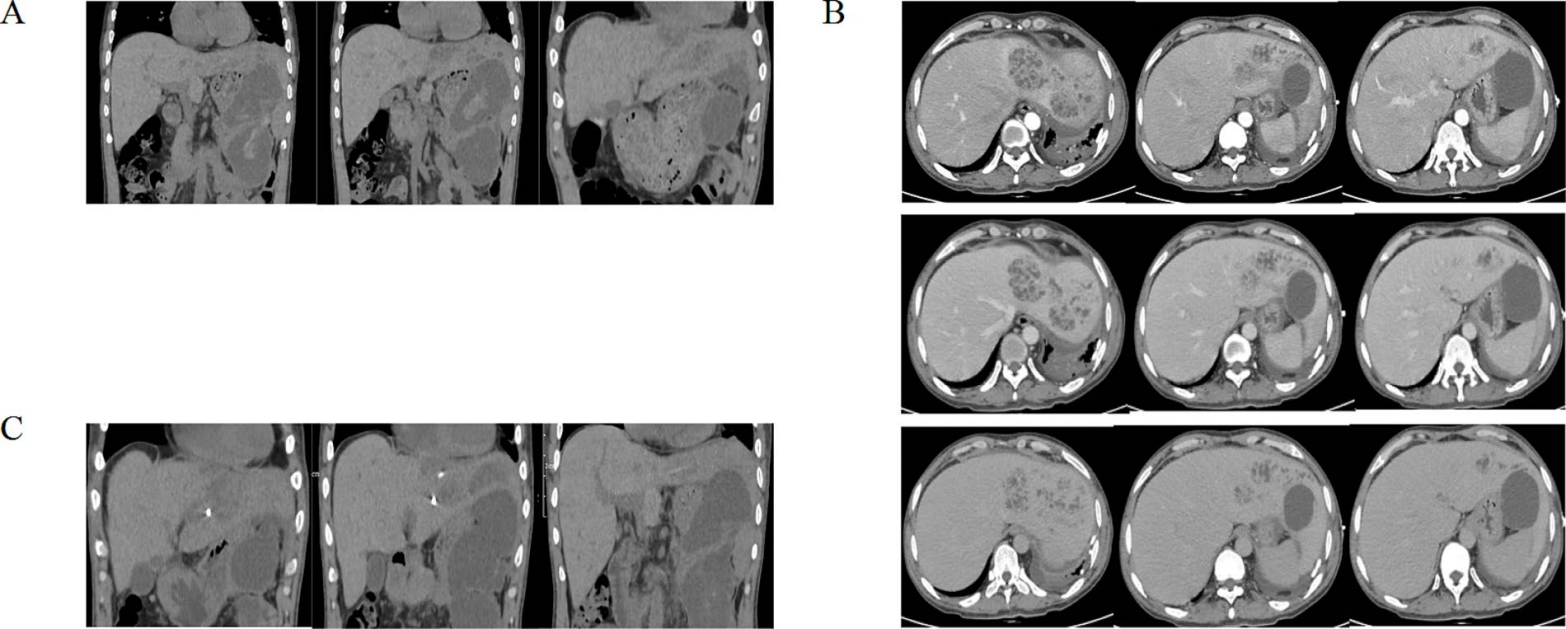
**(A)** Pre-admission whole abdominal CT revealed multiple space-occupying lesions in left hepatic lobe, mixed-density nodule in right subhepatic space, large space-occupying lesion in pancreatic tail and gallbladder stones. **(B)** Post-admission contrast-enhanced abdominal CT showed multiple abnormally enhancing cystic lesions in left hepatic lobe, consistent with infectious pathology, likely abscess formation; large pancreatic tail mass, likely benign, possibly pseudocyst formation with minor hemorrhage. **(C)** Follow-up CT after ultrasound-guided percutaneous catheter drainage of left hepatic abscess suggested pancreatic tail cyst with hemorrhage, and hepatic abscess suspected to communicate with pancreatic tail cyst or containing pancreatic secretions.

**Figure 2:**
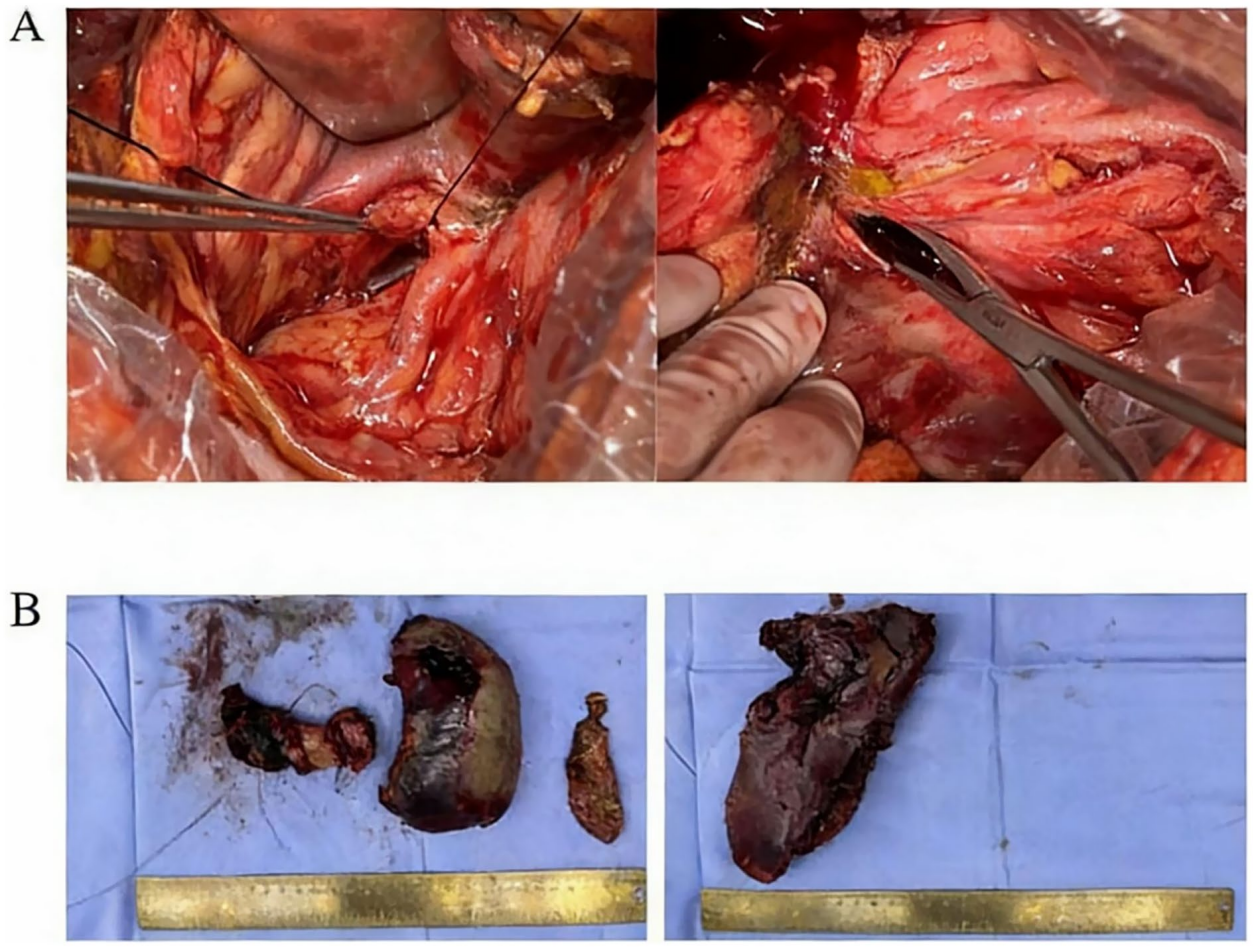
**(A)** Intraoperative images demonstrate a sinus tract between the pancreatic tail pseudocyst and the left hepatic lobe. **(B)** Surgical specimens are shown in the image. Left photograph demonstrates distal pancreas with adherent cyst wall, splenectomy specimen, and cholecystectomy specimen from left to right. Right photograph displays resected liver segments II with partial segment III.

**Figure 3:**
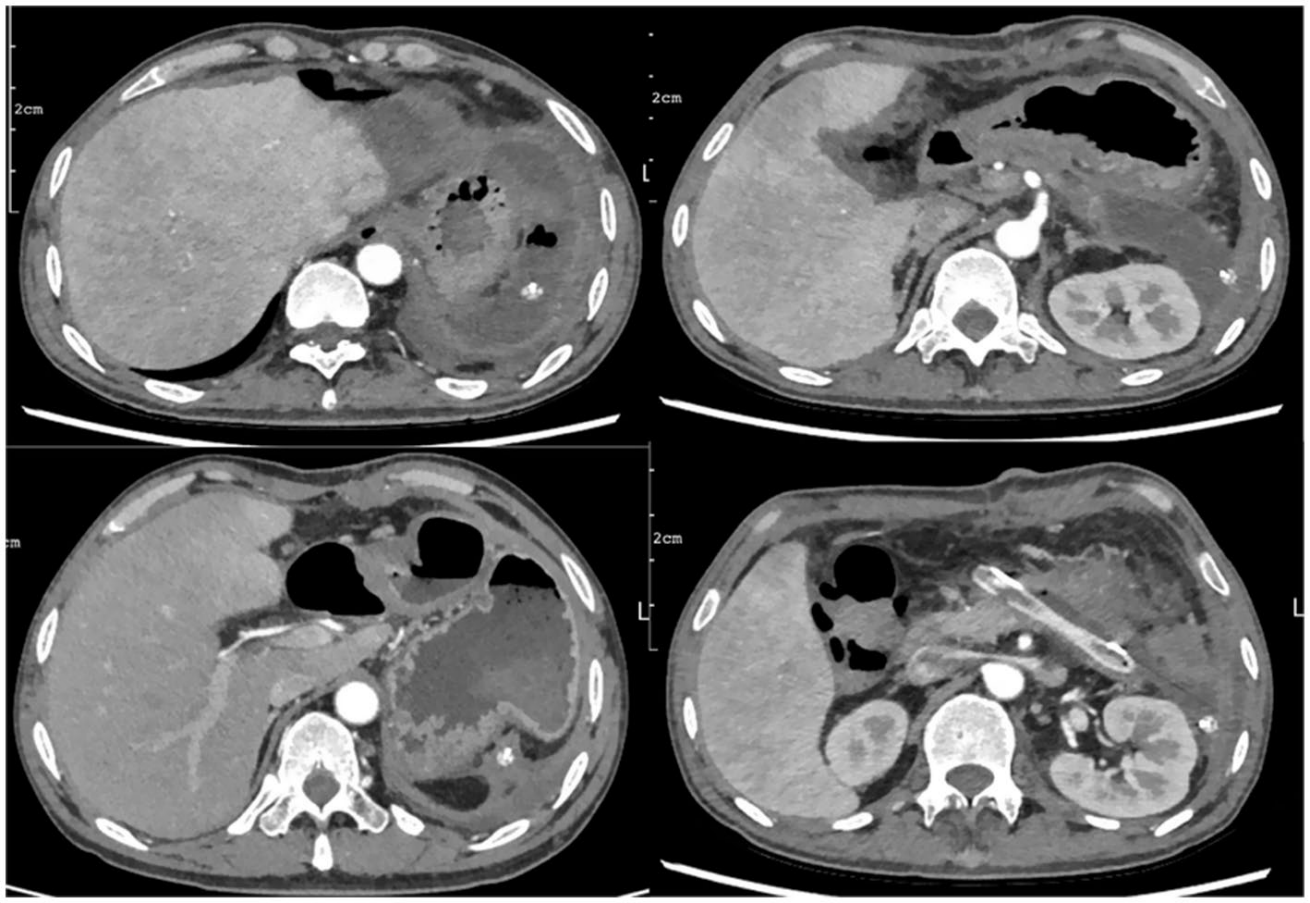
Postoperative CT demonstrates fluid collection at the surgical site with active drainage via the surgical drain.

**Figure 4:**
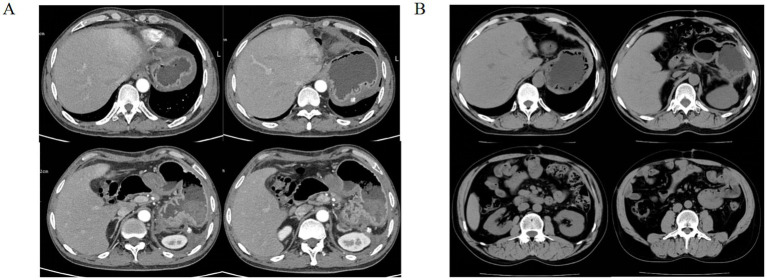
**(A)** Upper abdominal CT at 1 month after discharge demonstrated that the surgical drain could be safely removed. **(B)** Upper abdominal CT at 1 year after discharge revealed no obvious abnormality in the operative field.

A 43-year-old male patient presented to an outside hospital 4 months prior with upper abdominal distending pain radiating to the left mid-abdomen persisting for several hours. He was diagnosed with “acute pancreatitis with pancreatic pseudocyst” and underwent percutaneous drainage of the pancreatic pseudocyst without symptomatic improvement. Subsequently, the patient experienced chills and fever persisting for 6 h accompanied by severe left-sided abdominal pain, prompting presentation to our hospital. Past medical history was significant for previous acute pancreatitis and diabetes mellitus. Social history included daily cigarette smoking and alcohol abuse. Physical examination on admission revealed: temperature 36.7 °C, pulse 91 beats/min, respiratory rate 19 breaths/min, blood pressure 93/67 mmHg. Abdomen soft on palpation, with severe tenderness in the left abdomen without rebound tenderness or muscle guarding. A palpable mass approximately 10 cm × 8 cm was noted in the left abdomen, non-tender, with unclear borders, smooth surface, and no vascular bruits. Liver and spleen not palpable below costal margin, gallbladder not palpable, Murphy’s sign negative, no tenderness at McBurney’s point, shifting dullness negative. Laboratory findings: white blood cells 16.88 × 10^9^/L, neutrophil percentage 89.4%, C-reactive protein 63.69 mg/L, hemoglobin 68 g/L (Table 1). Whole abdominal computed tomography (CT) (Figure 1A) revealed gallbladder stones, multiple space-occupying lesions in the left hepatic lobe, and a large space-occupying lesion in the pancreatic tail. Post-admission, the patient received blood transfusion therapy and cefoperazone-sulbactam antibiotic therapy per IDSA guideline recommendations (5) without significant improvement. A repeat contrast-enhanced CT (Figure 1B) of the whole abdomen was subsequently performed. On contrast-enhanced CT, a mildly hyperattenuating component within the cyst was observed, consistent with intracystic hemorrhage. The patient received 2 units of RBC transfusion. Ultrasound-guided percutaneous catheter drainage of the left hepatic abscess was subsequently performed. Brown turbid fluid was drained post-procedure, with negative blood and drainage fluid cultures, and drainage fluid amylase level of 3786.9 U/L. The abscess cavity was evacuated by postoperative day 3, with minimal drainage output by day 5. Total drainage volume was 600 mL. Postoperatively, blood transfusion therapy continued with blood routine re-examination. Follow-up whole abdominal CT (Figure 1C) suggested pancreatic tail cyst with local complication of walled-off necrosis, and hepatic abscess suspected to communicate with pancreatic tail cyst or containing pancreatic secretions. Differential diagnosis was required from acute pancreatitis, pancreatic cancer with hemorrhage, and ruptured pancreatic cystic tumors with hemorrhage (Table 2). Based on whole abdominal CT findings, combined with the patient’s history of acute pancreatitis and pancreatic pseudocyst drainage, we confirmed the diagnosis of pancreatic tail cyst with local complication of walled-off pancreatic necrosis and rare complication of hepatic abscess. The patient underwent laparoscopic cholecystectomy combined with pancreatic tail and pseudocyst resection, partial hepatectomy, and total splenectomy (Figures 2A,B). Intraoperative findings confirmed communication between the pancreatic tail pseudocyst and hepatic abscess, where the pancreatic pseudocyst had eroded through hepatic tissue to form an intrahepatic abscess cavity. The operation was performed via an open approach. The estimated blood loss was 400 mL and the operative time was 180 min. Partial hepatectomy included segments II–III. Pathology showed that the cyst wall was consistent with a pseudocyst, and the resected liver tissue demonstrated multifocal necrosis with chronic inflammatory cell infiltration. Postoperatively, surgical site infection and pancreatic fistula at the pancreatic stump were managed with continuous irrigation and drainage through double-lumen tubes placed intraoperatively. Postoperative CT (Figure 3) was performed to assess recovery. Following combined postoperative nutritional support, antibiotic therapy, and blood transfusion, the patient resumed normal diet and activities on postoperative day 12 and was discharged with drainage tubes in place. One month post-discharge, following upper abdominal CT re-examination (Figure 4A), the intra-abdominal surgical drainage tubes were removed. At one-year follow-up, the patient maintained normal diet and activities without recurrence of abdominal pain or fever. The follow-up upper abdominal CT (Figure 4B) showed no significant abnormalities in the original surgical area.

**Table 1:** Admission laboratory results.

Basic laboratory indices	Value	Unit	Reference range
White blood cell count (WBC)	**16.88**	10*9	3.50–9.50
Red blood cell count (RBC)	**2.87**	10*12	3.80–5.10
Hemoglobin (HGB)	**81**	g/L	115–150
Hematocrit (HCT)	**0.24**	L/L	0.350–0.450
Platelet count (PLT)	233	10*9/L	125–350
Absolute neutrophil count	**13.68**	10*9/L	1.80–6.30
Absolute lymphocyte count	1.29	10*9/L	1.10–3.20
Absolute monocyte count	**0.93**	10*9/L	0.10–0.60
Potassium (K+)	4.60	mmol/L	3.50–5.30
Sodium (Na+)	141.72	mmol/L	137.0–147.0
Chloride (Cl-)	106.53	mmol/L	99.0–110.0
Aspartate aminotransferase (AST)	**102.17**	U/L	13.0–35.0
Alanine aminotransferase (ALT)	40.30	U/L	7.0–40.0
Gamma-glutamyl transferase (GGT)	26.70	U/L	7.0–45.0
Alkaline phosphatase (ALP)	58.16	U/L	35.0–100.0
Total protein	**47.05**	g/L	65.0–85.0
Albumin (ALB)	**29.10**	g/L	40.0–55.0
Globulin	**17.95**	g/L	20.0–40.0
Albumin/globulin ratio (A/G)	1.62		1.2–2.4
Total bilirubin	**24.07**	umol/L	0.0–21.0
Direct bilirubin	**10.45**	umol/L	0.0–6.8
Indirect bilirubin	13.62	umol/L	5.0–20.0
High-sensitivity C-reactive protein (hs-CRP)	**49.81**	mg/L	0–1.0
Microbial culture results			
Blood anaerobic culture	Negative		
Blood oxygen demand culture	Negative		
Liver abscess drainage fluid culture	Negative		

Clinical grouping: the results were organized based on basic laboratory indicators and microbial culture results to reflect systemic involvement and infection status, respectively. Abnormal values are shown in bold. Units and reference ranges are reported as provided by the testing laboratory; reference intervals may vary across institutions. “Negative” indicates no detectable antibody/serologic evidence in the reported assays.

**Table 2:** Differential diagnosis of hemorrhage in pancreatic pseudocysts.

Differential diagnosis	Support point	Counterpoint	
Pancreatic pseudocyst with hemorrhage	Severe upper abdominal pain. Nausea and vomiting. Elevated serum amylase/lipase. Abdominal enhanced CT suggests “active bleeding within a pseudocyst (contrast extravasation).”	Non	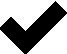
Acute pancreatitis	Severe upper abdominal pain. Nausea and vomiting. Elevated serum amylase/lipase.	A simple acute pancreatitis attack usually does not involve signs of acute or massive blood loss, such as a sudden drop in hemoglobin or shock.	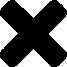
Pancreatic cancer with hemorrhage	When a pancreatic tumor undergoes necrosis and invades blood vessels, it can cause acute bleeding, severe upper abdominal pain, and nausea and vomiting, and it can also form retention cysts.	No progressive worsening jaundice/low back pain/sales. The tumor marker CA-199 was not elevated. Abdominal contrast-enhanced CT did not show solid pancreatic mass with vascular invasion.	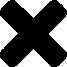
Ruptured and bleeding pancreatic cystic tumor	Mucinous cystadenomas/serous cystadenomas/intraductal papillary mucinous tumors, etc., can cause acute bleeding, severe upper abdominal pain, and nausea and vomiting if intracystic hemorrhage or rupture leads to hemoperitoneum.	Contrast-enhanced abdominal CT suggests that the cyst wall has uneven thickness, indicating inflammation, with no septations, wall nodules, or calcifications.	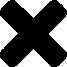

## Discussion

Pancreatic pseudocyst is well-recognized sequelae of pancreatitis (6). Most collections can be managed conservatively, whereas intervention is required when complications occur, such as infection, hemorrhage and rupture, or when drainage fails to achieve adequate source control (7).

Pancreatic-origin hepatic abscess is extremely rare. In our case, the lack of clinical improvement after percutaneous abscess drainage, together with markedly elevated amylase in the drainage fluid, suggested communication with a pancreatic collection. We therefore hypothesized direct penetration of an infected pancreatic pseudocyst through the hepatic capsule, and this was confirmed intraoperatively. A key concern in such cases is that exposure of pancreatic enzymes to bile may aggravate tissue injury and increase the risk of hemorrhagic complications.

Definitive management requires timely and sufficient source control (5). When percutaneous drainage and antibiotics are inadequate, particularly in the presence of a confirmed fistulous communication and ongoing risk of infection or bleeding, surgical treatment should be considered (8, 9). In this patient, laparoscopic cholecystectomy combined with pancreatic tail and pseudocyst resection, partial hepatectomy, and total splenectomy provided definitive removal of the infected cavity and the primary source, resulting in durable clinical resolution during follow-up.

Pancreatic-origin hepatic abscess presents challenges including difficult infection control, hemorrhage susceptibility, and disease recurrence. Percutaneous drainage and antibiotics show limited efficacy with frequent recurrence (10). Late-stage cyst enlargement causes intractable abdominal distension (11). Pancreatic-jejunal anastomosis following infection control cannot address clinical challenges of recurrent hepatic abscess hemorrhage from abnormal enzyme activation (12). This case innovatively employed partial hepatectomy combined with pancreatic pseudocyst resection and distal pancreatectomy with splenectomy. Despite operative complexity, thorough risk assessment and aggressive preoperative preparation comprehensively resolved these clinical challenges.

## Conclusion

This case report helps identify a rare subtype of hepatic abscess, providing new clinical evidence for its pathogenic mechanism such as direct rupture of pancreatic necrosis or infected pseudocyst into the liver. Through analysis of this case’s diagnostic and therapeutic course, we emphasize the necessity of screening for secondary hepatic infection in pancreatitis patients, offering clinicians a reference for recognition and treatment. Additionally, it provides a rare disease manifestation for differential diagnosis. This case demonstrates the diagnostic challenges arising from overlapping symptoms between pancreatitis and hepatic abscess highlighting the importance of combined radiological and clinical assessment, particularly the crucial role of contrast-enhanced CT in differentiating pancreatic lesions from hepatic abscesses (13). This case specifically elucidates the possibility of intracystic hemorrhage complicating pancreatic-origin hepatic abscess, offering a feasible therapeutic approach for clinical management. However, due to the rarity of pancreatic-origin hepatic abscess, individual case reports may not encompass all treatment modalities for future encounters with this disease. The diagnostic and therapeutic experience from this case such as combined surgical resection and drainage may not be applicable to all similar patients, necessitating strategy adjustments based on individual circumstances. The conclusions of this case are based on a single patient, and its pathogenic characteristics and surgical outcomes may be influenced by incidental factors, requiring validation through larger sample studies (14).

## Patient perspective

Initially, I only experienced upper abdominal discomfort and intermittent fever, which I dismissed as a common gastrointestinal issue and did not take seriously. Following admission and diagnostic workup, I was informed of a pancreatic-origin hepatic abscess with concurrent pancreatic cyst requiring surgical intervention. I was considerably apprehensive about the surgery; however, the medical team provided detailed explanations regarding the etiology, surgical necessity, associated risks, and alternative treatment options. Through thorough communication with myself and my family members, I was able to participate in the decision-making process and gradually felt reassured. During the early postoperative period, pain and weakness were significant, but under the care of the medical team with adequate analgesia, nutritional support, and rehabilitation guidance, I progressively recovered, with stable body temperature and symptom improvement. I now have a greater appreciation for the importance of adherence to scheduled follow-up appointments as advised by my physicians. The surgeon informed me that in his many years of clinical practice, he had never encountered such a case. I hope my experience can serve as a reference for the diagnosis and treatment of patients with similar conditions.

## Data Availability

The original contributions presented in the study are included in the article/Supplementary material, further inquiries can be directed to the corresponding author.
